# A clinical risk prediction model for perioperative lower extremity DVT in patients undergoing spinal fracture surgery

**DOI:** 10.3389/fsurg.2025.1597101

**Published:** 2025-09-18

**Authors:** ShuYuan Zhuang, Jing Wang, Peng Du, SiHong Dong, Jiao Wu, DeLong Li, YuanTong Zang, Li Li

**Affiliations:** ^1^ School of Nursing, Inner Mongolia Medical University, Hohhot, China; ^2^ Peking University Cancer Hospital (Inner Mongolia Campus) & Affiliated Cancer Hospital of Inner Mongolia Medical University, Hohhot, China; ^3^ Department of Spinal Surgery, The Second Affiliated Hospital of Inner Mongolia Medical University, Hohhot, China; ^4^ Department of Rehabilitation, The Second Affiliated Hospital of Inner Mongolia Medical University, Hohhot, China; ^5^ Nursing Department, The Second Affiliated Hospital of Inner Mongolia Medical University, Hohhot, China

**Keywords:** spinal fracture, deep vein thrombosis, risk factors, predictive model, lower limb

## Abstract

**Objective:**

To develop a perioperative lower-extremity deep vein thrombosis (DVT) risk prediction model for spinal fracture surgery patients using logistic regression, supporting clinical prevention strategies.

**Methods:**

Clinical data from 249 patients undergoing spinal fracture surgery (July 2019–October 2024) were retrospectively analyzed. Participants were divided into a model group (*n* = 166) and a validation group (*n* = 83) in a 2:1 ratio. Univariate and multivariate logistic regression identified independent risk factors for perioperative DVT, and a predictive model was established. Model fit was evaluated using the Hosmer-Lemeshow test, and predictive performance was assessed via receiver operating characteristic (ROC) curve analysis.

**Results:**

Independent risk factors included perioperative blood transfusion, elevated C-reactive protein, D-dimer >500 μg/L, hypertension, age ≥60 years, and prolonged bed rest. The model [*P* = 1/(1 + e^−Z)] demonstrated a good fit (Hosmer-Lemeshow *χ*^2^ = 12.139, *P* = 0.807). ROC analysis showed AUC values of 0.75 (95% CI: 0.80–0.92) for the model group and 0.81 (95% CI: 0.64–0.98) for the validation group, indicating robust predictive performance.

**Conclusion:**

The identified risk factors are critical predictors of perioperative DVT in spinal fracture patients. The proposed model exhibits strong clinical utility for early risk stratification and intervention guidance.

## 1 Introduction

Spinal fractures account for approximately 5% to 6% of all fractures (1), with the most common cause being high-energy trauma events. During the perioperative management of patients with spinal fractures, the risk of developing deep vein thrombosis (DVT) in the lower limbs shows a significant upward trend. DVT formation poses significant risks, potentially leading to delayed surgical treatment in the early stages, thereby prolonging the patient's hospital stay and significantly increasing medical costs. More seriously, if DVT is not managed promptly and effectively, it may progress to life-threatening pulmonary embolism (PE) (2). According to literature reports, the overall incidence rate of DVT following spinal surgery ranges from 0.2% to 31% (3). Based on the analysis of collected data, proactive prevention strategies have demonstrated significant clinical benefits in the prevention of DVT during the perioperative period in patients with spinal fractures. Although current orthopedic research on DVT in major surgeries has preliminarily identified some common risk factors (4), there remains a significant gap in research focused on specific risk indicators and targeted intervention measures during the perioperative period in patients with spinal fractures. First, there is a lack of risk indicators that account for dynamic changes during the perioperative period. Existing studies primarily focus on preoperative static factors (such as age, D-dimer levels, comorbidities, etc.), while neglecting the impact of perioperative dynamic indicators (such as surgical approach, intraoperative blood loss, postoperative bedrest duration, timing of anticoagulant initiation, etc.) on DVT occurrence (5); Second, there is no specific risk warning model established for the Chinese population. Existing DVT risk models (such as the Risk Assessment Profile) are primarily designed for Western trauma populations, and some key variables (such as post-discharge follow-up data) are difficult to obtain in real time in clinical practice, making them unsuitable for guiding preoperative decision-making (6). The incidence of DVT in Asian populations is significantly lower than in Western populations, and directly applying Western models may underestimate or overestimate actual risks (7). In particular, there is currently no multi-factor-defined, accurate, and targeted DVT risk warning model. This study employed a retrospective analysis method, systematically collected clinical case data from patients undergoing spinal fracture surgery. Using univariate screening and logistic multivariate regression models, we investigated the significant independent risk factors for DVT formation after spinal fracture surgery. Based on this, we constructed a DVT risk warning model based on logistic risk regression using standardized modeling formulas to achieve rapid and accurate screening of the risk of lower extremity DVT in patients undergoing spinal fracture surgery. The specific findings are reported as follows:

## 2 Information and methods

### 2.1 Research data

Clinical data from 249 patients with spinal fractures who underwent surgical treatment at a tertiary orthopedic specialist hospital in Inner Mongolia from July 2019 to October 2024 were retrospectively collected and analyzed. These patients were divided into a model group (*n* = 166) and a validation group (*n* = 83) in a 2:1 ratio to ensure the statistical validity of the model construction and validation process through this grouping. The patients were 18–76 years old, with a mean age of 58.65 ± 7.49 years and a BMI of 25.16 ± 4.40 kg/m^2^. The inclusion criteria were as follows: (1) age ≥18 years; (2) complete clinical data; (3) no history of thrombosis or other related diseases before admission; (4) clear consciousness, normal cognitive function, and ability to cooperate with the investigations and follow-up; and (5) color ultrasound of the venous vessels of the lower limbs. Complete data. The exclusion criteria were as follows: (1) diagnosis of a malignant tumor or spinal fracture caused by osteoporosis or pathological spinal fracture; (2) severe varicose veins, coagulation dysfunction, or a history of oral anticoagulants; (3) orthopedic pathology in parts of the body other than the spine; and (4) any other major stressful events during the period of admission to the hospital. The Declaration of Helsinki and all methods were approved by the Ethics Committee of the Second Affiliated Hospital of Inner Mongolia Medical University (Number: EFY20240059).

### 2.2 Research methodology

#### 2.2.1 Data collection

A general information questionnaire was developed to record detailed information on the selected patients' demographics, comorbidities, treatments, and major laboratory parameters. Specific information included sex, age, body mass index (BMI), history of preexisting medical conditions, the specific site of the fracture, American Society of Anesthesiologists (ASA) classification, the time interval between fracture and surgery, postoperative bed rest, length of surgical operation, history of smoking, and history of perioperative blood transfusion; intraoperative hypothermia, postoperative drainage, D-dimer (D-D), C-reactive protein (CRP), D-D, and C-reactive protein (CRP) levels were recorded on the first day of the operation; and whether intraoperative hypothermia, postoperative drainage, and D-D levels were recorded on the first day of the operation. D-D, C-reactive protein (CRP), hemoglobin (HGB), cholesterol concentration (CHOL), procalcitonin concentration (PCT), and fibrinogen concentration (FPC).), fibrinogen concentration (FIB), prothrombin time (PT), activated partial thromboplastin time (APTT), and hypoproteinemia (TP concentration ≤ 60 g/L or ALB concentration ≤ 35 g/L).

#### 2.2.2 Research methods

The perioperative course and treatment of the patients were meticulously documented. Based on imaging examination results, such as color Doppler ultrasound and the diagnostic criteria for DVT, the model group (*n* = 166) was screened for DVT, with positive cases assigned to the DVT group. Univariate analysis was performed to compare the variability in baseline characteristics between the two model group subgroups: DVT group and non-DVT groups. Variables demonstrating statistically significant differences in the univariate analysis were incorporated into a multivariate logistic regression model to identify independent risk factors for lower limb deep vein thrombosis (DVT) in spinal fracture patients during the perioperative period. A perioperative DVT risk prediction model was developed based on the key variables identified in this model. The Hosmer-Lemeshow test was employed to assess the consistency between the model predictions and actual observations to evaluate the prediction model's performance. Additionally, the area under the curve (AUC) was calculated using a receiver operating characteristic (ROC) curve to quantify the model's predictive efficacy and classification ability. Furthermore, venous Doppler ultrasonography, a noninvasive and user-friendly imaging technique, is widely recognized in clinical practice because of its high sensitivity (>90%) and specificity (>90%) for diagnosing DVT (8). Therefore, the preoperative and postoperative venous ultrasonography results were utilized as an objective diagnostic basis for DVT in this study, ensuring the reliability and consistency of the data.

### 2.3 Statistical methods

The relevant analyses were performed using the SPSS 27.0 software package, customarily distributed measurements were described as (*x* ± *s*), and comparisons were made using the independent samples t-test. Data that did not conform to normally distributed measures were described as medians [P25, P75], and the rank sum test comparisons were made. Count data were described by the constitutive ratio (n[%]), and the chi-square test was performed. A logistic regression prediction model was constructed after independent risk factors were identified via multifactorial logistic regression analysis. The risk regression equation was *P* = 1/(1 + e^–Z). Note: *P* = probability of occurrence; *Z* = weighted sum of multiple independent variables,and the known constant *e* ≈ 2.718. In this study, the Hosmer-Lemeshow test was implemented to assess the calibration performance of the constructed model. With the help of plotting the subjects' work characteristics (ROC) curves and calculating their area under the curve (AUC), the discriminative and predictive efficacies of the model in distinguishing between different categories of objects were systematically assessed. The optimal cut-off value was determined based on the principle of maximization of the Jordon index, which was subsequently applied to set the predictive threshold of the model. This strategy aims to precisely delineate the diagnostic boundaries between sensitivity and specificity to optimize the decision-making accuracy. Statistical significance was indicated by a *p*-value of 0.05.

## 3 Results

### 3.1 Univariate analysis of perioperative lower extremity deep vein thrombosis in spinal fracture surgery patients in the model group

A total of 29 cases of perioperative lower extremity deep vein thrombosis occurred in 166 patients who underwent spinal fracture surgery in the model group. The incidence of DVT was 17.46% (29/166), with the above patients classified in the DVT group (*n* = 29), and the remaining patients were classified in the non-DVT group (*n* = 137). Univariate analysis within the model group revealed the differences between the two subgroups of the DVT and non-DVT groups in terms of information (indexes) such as age, duration of surgery, time in bed, history of hypertension, history of coronary artery disease, history of perioperative blood transfusion, amount of postoperative drainage, whether or not hypothermia occurred intraoperatively, and the incidence of hypoproteinemia, D-dimer, and C-reactive protein levels on the first postoperative day was statistically significant (*P* < 0.05). The details are listed in Table 1.

**Table 1:** Univariate analysis of perioperative lower extremity deep vein thrombosis in patients in the model group.

Variant		DVT group (29 cases)	Non-DVT group (137 cases)	*χ^2^*/*t*/*Z*	*P* value
Age (year)	≥60	24 (82.8)	66 (48.2)	11.532	0.001
	<60	5 (17.2)	71 (51.8)		
Sex	Male	17 (58.6)	79 (57.7)	0.013	0.909
	Female	12 (41.4)	58 (42.3)		
BMI (kg/m^2^)		24.89 ± 4.80	25.22 ± 5.01		
Fracture site	Cervical spine Thoracic spine	5 (17.2)	32 (23.4)	1.485	0.138
	Cervical spine Lumbar spine	4 (13.8)	20 (14.6)		
	Cervical, thoracic and lumbar spine	1 (3.4)	6 (4.4)		
	Thoracic spine Lumbar spine	6 (20.7)	27 (19.7)		
	Cervical spine	4 (13.8)	25 (18.2)		
	Thoracic spine	4 (13.8)	17 (12.4)		
	Lumbar spine	5 (17.2)	10 (7.3)		
ASA grade	Level II	7 (24.1)	38 (27.7)	3.750	0.053
	Level III	22 (75.9)	99 (72.3)		
Fracture to surgery time(d)		6 (3, 11)	6 (3, 9)	−0.371	0.711
Surgery time(min)		270 (185, 350)	200 (60, 385)	−2.627	0.009
Time in bed(d)		14 (13, 18)	8 (7, 11)	−8.218	0.000
History of hypertension	Yes	23 (79.3)	39 (28.5)	22.756	0.000
	No	6 (20.7)	98 (71.5)		
History of diabetes	Yes	7 (24.1)	27 (19.7)	0.081	0.776
	No	22 (75.9)	110 (80.3)		
History of coronary heart disease	Yes	17 (58.6)	43 (31.4)	6.556	0.010
	No	12 (41.4)	94 (68.6)		
History of perioperative blood transfusion	Yes	27 (93.1)	46 (33.6)	32.049	0.000
	No	2 (6.9)	91 (66.4)		
Postoperative drainage(ml)	≤100 ml	6 (20.7)	58 (42.3)	3.864	0.049
	>100 ml	23 (79.3)	79 (57.7)		
Whether hypothermia occurs intraoperatively	Yes	19 (65.5)	50 (36.5)	7.147	0.008
	No	10 (34.5)	87 (63.5)		
Hypoproteinemia	Yes	20 (68.9)	51 (37.2)	8.596	0.003
	No	9 (31.1)	86 (62.8)		
PT(s)	≤11 s	22 (75.9)	97 (70.8)	0.104	0.747
	>11 s	7 (24.1)	40 (29.2)		
APTT(min)	≤28 min	23 (79.3)	89 (64.9)	1.638	0.201
	>28 min	6 (20.7)	48 (35.1)		
D-Dimer(μg/L)	≤500 μg/L	2 (6.9)	61 (44.51)	12.838	0.000
	>500 μg/L	27 (93.1)	76 (55.5)		
FIB(g/L)	>3.5 g/L	17 (58.6)	52 (37.9)	3.400	0.065
	≤3.5 g/L	12 (41.4)	85 (62.1)		
CRP(mg/L)	>6.0 mg/L	23(79.3)	40(29.2)	23.441	0.000
	≤6.0 mg/L	6(20.7)	97(70.8)		

### 3.2 Multifactorial analysis of perioperative lower-limb deep vein thrombosis in spinal fracture surgery patients in the model group

In this study, variables showing statistical significance in univariate analyses, including age, length of surgery, bed rest cycle, history of hypertension and coronary heart disease, perioperative blood transfusion, postoperative drainage, intraoperative hypothermic events, hypoproteinemia status, D-dimer and C-reactive protein levels on the first postoperative day were entered into logistic multivariate regression models as covariates to identify the independent risk factors for lower extremity deep vein thrombosis (DVT) after spinal fracture. (The categorical variables in the above indicators were first assigned values when used as independent variables; the assignment methods are specified in Table 2.) The results of the logistic regression analysis revealed that a history of perioperative blood transfusion, a high level of C-reactive protein (CRP), a D-dimer concentration >500 μg/L, a history of hypertension, an age ≥60 years old, and a prolonged bed rest period were independent risk factors for perioperative lower-extremity DVT in spine fracture patients. Detailed results are presented in Table 3.

**Table 2:** Assignment methods for the categorical variables.

Variable item	Assignment method
Age	<60 = 0, ≥60 = 1
History of hypertension	No = 0, Yes = 1
History of coronary heart disease	No = 0, Yes = 1
History of perioperative blood transfusion	No = 0, Yes = 1
Postoperative drainage	≤100 ml = 0, >100 ml = 1
Whether hypothermia occurs intraoperatively	No = 0, Yes = 1
Hypoproteinemia	No = 0, Yes = 1
D-dimer	≤500 μg/L = 0, >500 μg/L = 1
C-reactive protein	≤6.0 mg/L = 0, >6.0 mg/L = 1

**Table 3:** Logistic regression analysis of perioperative lower-limb deep vein thrombosis in the model group.

Risk factor	*β*	SE	Wald *χ*^2^	*P* value	OR	95% CI
Constant term	−20.480	4.467	21.016	0.000	–	–
History of hypertension	2.169	0.743	8.520	0.003	8.758	2.039∼30.543
CRP>6.0 mg/L	3.927	0.737	20.769	0.000	42.754	10.039∼101.543
Age ≥ 60	1.730	0.622	7.737	0.004	5.739	1.667∼19.076
D-dimer>500 μg/L	2.425	0.742	10.675	0.001	11.307	2.639∼48.456
History of perioperative blood transfusion	4.513	0.769	27.769	0.000	45.602	11.924∼115.708
Prolonged bedtime	1.081	0.429	7.344	0.002	3.948	1.971∼7.837

### 3.3 Construction and testing of an early warning model for perioperative DVT risk in patients undergoing spinal fracture surgery

#### 3.3.1 Establishment of early warning model

Based on the independent risk factors established by logistic risk regression analysis, namely, a history of perioperative blood transfusion, a high level of C-reactive protein (CRP), a D-dimer concentration >500 μg/L, a history of hypertension, an age ≥60 years, and prolonged bed rest, a total of six items were given the sequential numbers of X1–X6 to complete the extraction of partial regression coefficients of the predictors, which described the perioperative DVT risk warning model for patients who underwent spinal fracture. The logistic regression linear equation of the early warning model for perioperative DVT risk was as follows: Logit(*p*) = −20.480 + 4.513 × X1 + 3.927 ×  X2 + 2.425 × X3 + 2.169 × X4 + 1.730 × X5 + 1.081 × X6.

#### 3.3.2 Goodness-of-fit test of the early warning model

In this study, the Hosmer-Lemeshow goodness-of-fit test was used to judge the fit of the model; the results showed *χ*^2^ = 12.139, *P* = 0.807, which revealed that the difference between the observed values and the predicted values of the model was not significant, suggesting that there was no significant bias in the model, and that the fit was good.

#### 3.4.2 Differentiation test of early warning model

**Figure 1:**
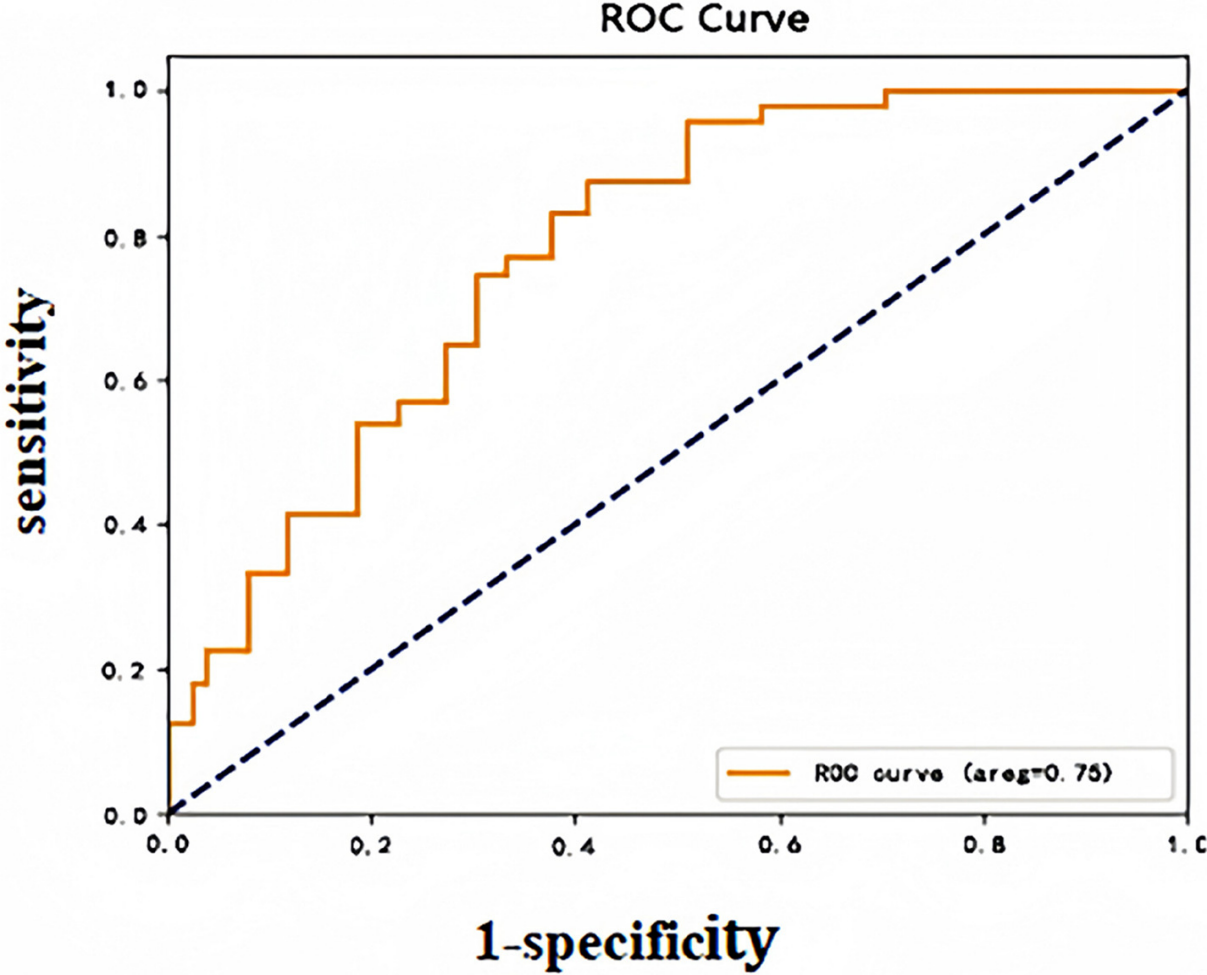
Early warning model ROC curve (modeling group).

In this study, after importing the clinical case data from the model group into the prediction model, the predictive probability value of each sample was calculated and this probability was used as a predictor variable. The occurrence status of DVT was defined as a dichotomous response variable. The ROC curves of the subjects were plotted, and the model's classification performance was quantitatively assessed by calculating the area under the curve (AUC). The Yoden index maximization criterion was further used to determine the optimal cut-off value for the model prediction probability, which corresponded to a model prediction threshold of 0.076, an AUC of 0.75 (95% CI: 0.80–0.92), a sensitivity of 82.75%, and a specificity of 75.9%, which confirms that the model had a good predictive ability. The ROC curves of the model group are plotted in Figure 1.

### 3.5 Validation of early warning model

**Figure 2:**
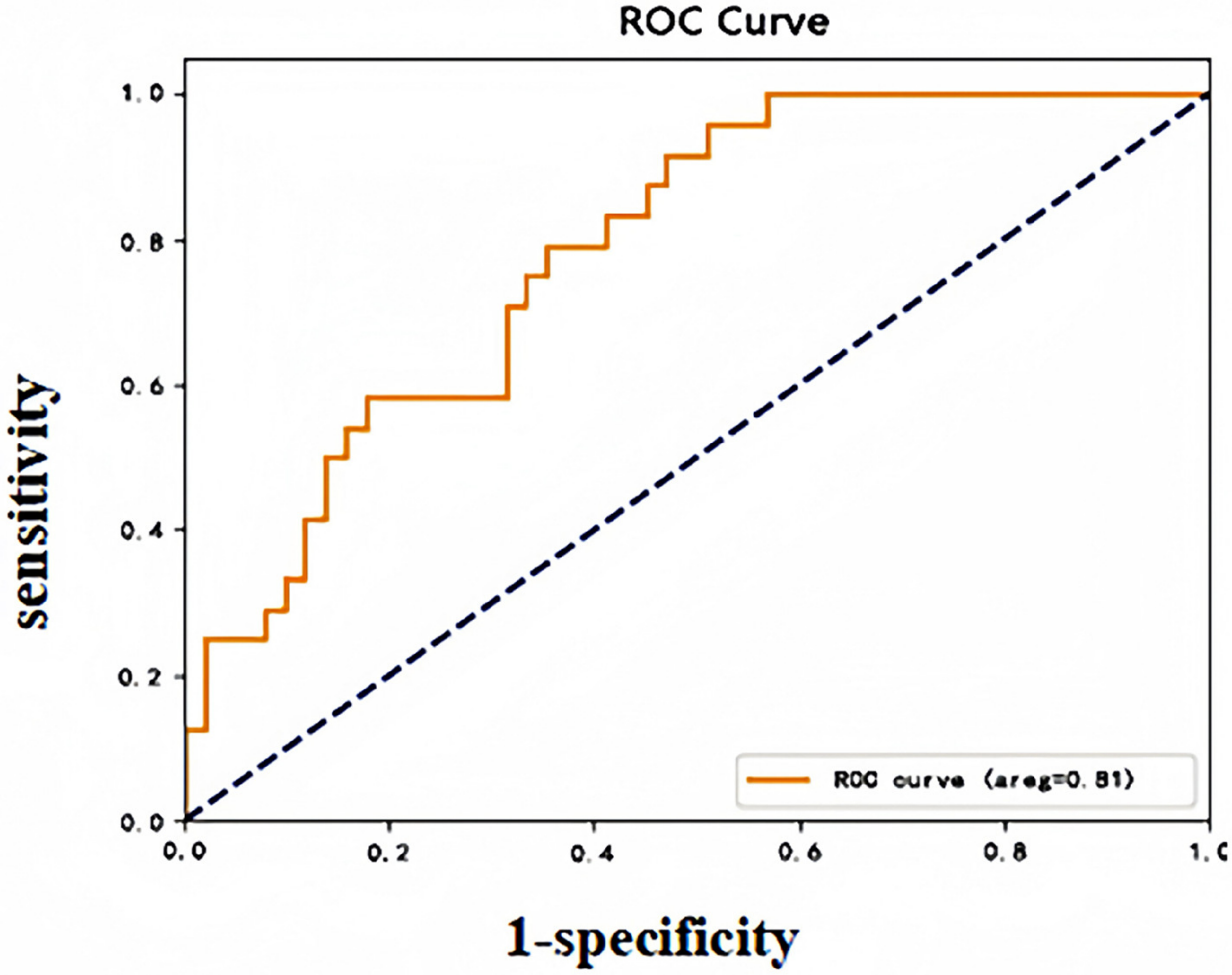
Early warning model ROC curve (validation group).

DVT occurred in 11 of 83 patients in the validation group during the perioperative period, and the early warning results revealed a sensitivity of 72.73%, a specificity of 84.72%, and an accuracy of 83.13%. The validation group early warning model subject operating characteristic (ROC) curve (see Figure 2), AUC: 0.81 (95% CI: 0.64–0.98), indicated that the model risk warning ability was good.

## 4 Discussion

The results of this study revealed that the incidence of perioperative lower extremity deep vein thrombosis in patients with spinal fractures was 17.46%, which is an approximate or slightly lower incidence than the epidemiological data reported in the literature (0.2%–31%) (9, 10). However, this result still suggests that the potential risk of perioperative DVT in such patients needs to be given due attention in the clinic. Notably, perioperative hemodynamic and venous haemodynamic abnormalities in patients with thoracolumbar spine fractures caused by high-energy injuries are more pronounced because of the complex mechanism of trauma and the high demand for tissue repair, further exacerbating the formation of DVTs. In clinical therapeutic interventions for spinal fractures, establishing a risk warning model to quickly and accurately assess the risk of DVT and provide patients with individualized preventive and therapeutic measures is an urgent problem. We conducted a retrospective study of patients admitted for spinal fracture surgery in recent years to systematically assess a series of potential high-risk factors associated with postoperative DVT formation. Univariate and logistic multifactorial analyses were performed on all suspected high-risk factors closely related to perioperative DVT, based on which a risk warning system was constructed via logistic regression modeling to provide a more precise and effective strategy for preventing DVT in major orthopedic surgeries.

In this study, a history of perioperative blood transfusion, high levels of C-reactive protein (CRP), D-dimer concentrations >500 μg/L, a history of hypertension, age ≥60 years, and prolonged bed rest were identified by univariate and multivariate logistic regression analyses as independent risk factors for perioperative lower-extremity DVT in patients with spinal fractures. Previous studies have shown that perioperative blood transfusion during surgery is strongly associated with thrombosis (11, 12). A multicenter study conducted by a national scholar (13) confirmed that intraoperative and/or postoperative blood transfusion, as well as postoperative blood transfusion alone, were significantly associated with the development of DVT in patients with traumatic spinal fractures, and the results of the present study are consistent with this finding. The mechanisms by which perioperative blood transfusion increases the risk of DVT may involve a hypercoagulable state of the blood, vascular endothelial injury, and abnormalities in immune regulation (14). When red blood cells (RBCs) or plasma are transfused, coagulation factors in blood products may lead to increased erythrocyte aggregation and decreased cell membrane deformity, thereby triggering a hypercoagulable state in the blood (15). In addition, the transfusion process may trigger an inflammatory response, leading to vascular endothelial damage and hemodynamic alterations. It may affect the body's immunomodulatory function, increasing the risk of thrombosis. Findings have shown that C-reactive protein (CRP), an acute temporal response protein with low serum levels in healthy individuals, is significantly elevated to reflect the presence of a systemic sensitively, but not specific, inflammatory response state in the organism. Studies have shown (16) that the expression level of CRP is significantly correlated with the degree of activity of the inflammatory response, especially in the state of trauma or infection, and its dynamic changes can provide an objective basis for the quantitative assessment of the inflammatory state. Previous retrospective cohort studies have shown that the inflammatory response is one of the most important factors in thrombosis and can be regarded as a chronic, low-level sterile inflammatory process (17). Individuals with high C-reactive protein levels have a significantly increased risk of developing DVT during the perioperative period, suggesting that C-reactive protein (CRP) stimulates the production of tissue factor (TF) by monocytes, which directly triggers abnormalities in coagulation mechanisms and promotes platelet adhesion and aggregation, ultimately leading to thrombosis. In addition, elevated CRP levels are positively correlated with the degree of complement system activation, further exacerbating vascular endothelial damage and promoting thrombus formation and development (18–20). Therefore, continuous monitoring of C-reactive protein concentration is highly valuable for the early identification of patients at high risk of DVT, especially those undergoing different orthopedic surgery.

As a specific marker of the fibrinolytic process, an increase in D-dimer concentration directly reflects the presence of hypercoagulability and the subsequent enhancement of fibrinolytic activity *in vivo*, which is closely related to thrombosis and its induced dysregulation of the dynamic balance between coagulation and fibrinolysis (21). According to the available literature (22), activation of the fibrinolytic system is triggered when a thrombus is present in the body, a process in which cross-linked fibrin is broken down to produce derivatives such as D-dimer. Hui et al. (23) reported that the sensitivity and specificity for predicting DVT with an elevated D-dimer level on day 3 after spinal surgery were 72.7% and 76.5%, respectively, and the D-dimer cut-off level was 5.82 μg/ml. Joseph et al. (24) reported that surgery increased D-dimer levels in patients with spinal fractures combined with spinal cord injury. When D-dimer concentrations were ≥2 times the normal's upper limit, their DVT risk was 3.5 times greater than that of patients with typical D-dimer values. The present study revealed that D-dimer levels fluctuate and increase after surgery in patients with spinal fractures. Elevated D-dimer levels can reflect the status of thrombosis in the body. A D-dimer level >500 μg/L may indicate the presence of DVT, and the results of the present study are basically in agreement with those of the above studies. This study confirmed that there is a close relationship between a history of hypertension and the risk of DVT in patients undergoing spinal fracture surgery. Most scholars believe prolonged elevated blood pressure damages vascular endothelial cells, an important factor in developing DVT (25). Second, hypertension is often associated with increased red blood cell aggregation and increased plasma viscosity, coupled with decreased vascular elasticity and luminal narrowing, which trigger the body's coagulation cascade response, which in turn significantly elevates the risk of DVT (26). The present study revealed that age greater than or equal to sixty years and prolonged bed rest were independent risk factors for DVT in patients with spinal fractures and that these factors can be used together as indicators to assess the risk of DVT. Prolonged bed rest increases the risk of DVT after orthopedic surgery. Reduced activity after orthopedic surgery, decreased cardiac output, decreased pump function of the lower limb muscles, increased resistance to venous blood return in the lower limbs, and activation of the coagulation state are more likely to lead to DVT (27). Spinal fractures are unique compared with other fractures, especially when combined with spinal cord and nerve damage, sensory and motor dysfunction of the lower limbs, and prolonged bed rest is more common in these patients after surgery, which can further exacerbate venous blood flow stagnation and weaken muscle pump function, and therefore the incidence of DVT is even greater. Previous studies have established advanced age as a risk factor for DVT in patients undergoing major orthopedic surgery and noted that this risk tends to increase with age (28). This is mainly because changes in vascular fragility are positively correlated with age, and the older the patient is, the poorer the cardiac function, the greater the fibrin activity, the greater the platelet aggregation, and the more severe the intima-media damage and blood flow abnormalities in his/her vessels (29). In addition, advanced age usually coexists with other DVT risk factors (e.g., obesity, hypertension, and prolonged bed rest), and there is a synergistic effect between these risk factors, which can further increase the risk of DVT.

## 5 Conclusions

In this study, based on the results of a multivariate regression analysis model (OR values and their influence weights), an early warning model for early risk identification of perioperative DVT in patients undergoing spinal fracture surgery (incorporating a total of six independent risk factors) was further developed through data processing with logistic regression formulas. Using the Hosmer-Lemeshow test, we validated the predictive efficacy of this predictive model in terms of the number of perioperative DVT cases, and the results showed consistency between its predictive values and the actual number of observed cases. A good fit (*χ*^2^ = 12.139, *P* = 0.807) suggests that the early warning ability of the model has some credibility and is clinically beneficial. This study further validated the logistic regression model internally and externally. The AUC, sensitivity, and specificity of the model for predicting the perioperative occurrence of DVT in patients who underwent spinal fracture surgery in the model group, as plotted by the subjects' operating characteristic (ROC) curves, were 0.75%, 82.75%, and 75.9%, respectively. The validation group's values were 0.75%, 82.75%, and 75.9%, respectively. Moreover, specificity was 0.81, 72.73%, and 84.72%, respectively, and the best diagnostic value was obtained according to the maximum principle of Jordon's index, with a *p*-value of 0.075, that is, when the predictive probability was ≥0.075, suggesting that perioperative patients are at risk of developing DVT. The above data revealed that the sensitivity and specificity were significantly more significant than the threshold of 70% in the constructed model and validation phase, which indicated that the developed model demonstrated a strong differentiation ability to identify risk factors and that the probability of appearing against false-negative and false-positive cases was low. These results suggested that the early warning model had good clinical predictive efficacy and applicability, which is beneficial for clinical operation and application.

In this study, unifactorial and multifactorial logistic regression analyses were used to identify and quantify risk variables that contribute significantly and independently to the development of DVT as a clinical event in patients undergoing spinal fracture surgery during postoperative recovery. The model considers a variety of factors that may influence the development of DVT, including a history of blood transfusion, medical history, age, coagulation, and inflammatory markers. The study's results revealed that the constructed early warning model had a high AUC in both the model and validation groups, and the evaluation indices, such as sensitivity and specificity, were within the acceptable range. This study suggested that the model demonstrated excellent predictive performance and accurately quantified the likelihood of patients undergoing spinal fracture surgery to develop lower-limb DVT, thus providing strong support for decision-making in clinical practice. Notably, although this study achieved its intended goals and produced valuable findings, its results are subject to several limitations that need to be fully considered when interpreting the research results are interpreted. For example, the sample sizes included in the retrospective analyses tend to be more limited, which may limit the ability of the constructed models to be generalized, that is, the efficacy of the model's performance in the face of unseen data. Notably, there is significant heterogeneity in treatment protocols and patient composition across different healthcare organizations. This implies that the existing models' scope of application and generalizability must be more fully explored and confirmed through subsequent studies. In the future, we will continue to monitor the research progress in this area and continuously improve and update such risk warning models.

## Data Availability

The raw data supporting the conclusions of this article will be made available by the authors, without undue reservation.
